# Relational encoding promotes creative insight for problem-solving

**DOI:** 10.3758/s13421-025-01685-1

**Published:** 2025-01-24

**Authors:** Kenneth J. Kurtz, Leif Haley, Alexus Longo, Shanti Astra, Hannah Meltzer, Gavin Suwara, John D. Patterson

**Affiliations:** 1 Department of Psychology, Binghamton University, PO Box 6000, Binghamton, NY 13902 USA; 2 Department of Psychology, Pennsylvania State University, State College, PA 16801 USA; 3 Department of Psychology, University of Wisconsin-Madison, Madison, WI 53706 USA

**Keywords:** Insight problem-solving, Problem encoding, Relational encoding, Creative cognition, Context-dependent properties

## Abstract

The nature and basis of creative thought has been the subject of wide-ranging inquiry. It is well established that people tend to struggle to solve problems that require an insight—and that this limitation is not readily alleviated. What can help produce more successful creative cognition? We propose a benefit from increased focus on the relations that hold between the elements of a problem situation. The present research addresses a novel technique to promote relational encoding by guiding participants to generate relational content connecting problem elements. In two experiments, we find that participants who engage in relational encoding are more likely to solve insight problems than controls. Theoretical and applied implications of the findings are addressed.

**Supplementary Information:**

The online version contains supplementary material available at 10.3758/s13421-025-01685-1.

One of the frontiers in the scientific study of human thinking is creative problem-solving (i.e., the ability to discover via insight or analysis a viable solution to a problem that lacks an evident solution or solution strategy). From the perspective of psychological explanation, we would like to know how this type of problem-solving works and why it is so cognitively challenging. From a broader and more applied perspective, we would like to know how to help people solve the difficult problems that arise in daily life, in professional work, and at the level of society as a whole. We focus on what are known as “insight problems” which are typically 1) fairly difficult to solve because they are not amenable to a systematic strategy or standardized solution approach and 2) solved in a manner that involves seeing or thinking about the problem content in a different way. To be clear, a neighboring body of research addresses the phenomenology (i.e., the dynamics or qualitative sense) of insight experiences (see Salvi et al., 2024), but in the present work we focus on creative cognition as it relates to solving insight tasks where the term “insight” refers to the nature of the problem/solution rather than the phenomenological nature of the experience.

Our theoretical proposal draws on two important threads in the existing problem-solving literature and ties them together in a new and potentially impactful manner. The first thread is the importance of problem representation (i.e., how the way in which the provided content of the problem is psychologically encoded may determine success to a greater degree than the procedures or abilities brought to bear). The second thread is the structural alignment process underlying analogical problem-solving (i.e., seeing a target problem as structurally similar to a familiar source can lead to a candidate solution). The new direction that guides our present research is the proposal that the structural (relational) representations usually considered for their role in analogy also matter critically to problem-solving when there is no comparison process or analogy-making involved at all. We apply relational encoding instead to the issue of how a problem is represented—we propose that an important way to build or restructure a problem representation that supports problem-solving success is to maximize the representational commitment to the relations that hold between problem elements.

Toward elucidating this approach, we begin with some relevant background. Psychological research has addressed the question of insight-based problem-solving from early Gestalt approaches (Duncker, 1945; Kohler, 1925; Maier, 1931) to the contemporary (e.g., Ohlsson, 2011; Weisberg, 2006). There appears to be relatively broad agreement that Duncker’s (1945) construct of functional fixedness is a key explanatory component—people habitually construe the elements of a problem situation in terms of past experience or canonical semantic roles and then have difficulty thinking about them in other ways. A major contribution that has emerged is that the initial encoding or problem representation strongly mediates problem-solving success (e.g., Kaplan & Simon, 1990; Knoblich et al., 1999; Ohlsson, 1992). Specifically, functional fixedness can be seen as an unquestioned commitment to an initial “take” on the problem situation. By altering the presentation of the problem or by promoting reinterpretation, it is possible to alter problem-solving outcomes.

Patrick and Ahmed (2014) discuss three different paths that have been studied to promote successful solutions to insight problems. The first is incubation, whereby the problem-solver may escape being stuck by taking time away from actively considering the problem (see Sio & Ormerod, 2009). The second is providing hints (though to the extent that such hints make the problem easier to solve, this seems outside the scope of actually improving problem-solving ability). The third is providing training in how to approach this particular type of problem-solving challenge. Ohlsson’s (1992) representation change theory specifically suggests that an impasse in problem-solving can be resolved by restructuring the problem through redescribing its elements, finding new elements to encode, or relaxing constraints in the formulation of the goal. This theoretical motivation has provided the foundation for a number of training approaches.

Patrick and Ahmed (2014) developed a training technique based on first explaining functional fixedness and then teaching people a process for individually considering each problem element and listing its possible functions. Chrysikou (2006) was motivated by work on goal-derived categories (Barsalou, 1991) to develop a training technique in which people learn to list alternative categories for objects. McCaffrey (2012) introduced a specific claim about the type of representation change that is required to overcome functional fixedness—namely, the noticing of obscure features of objects in a problem setting. McCaffrey (2012) developed and demonstrated benefits due to a *generic parts training* technique, in which participants learn to decompose objects into parts in order to reveal features not linked to objects’ traditional functions.

In the present research we build on this tradition with two major departures. The more practical issue is that, rather than putting participants through an intensive training regimen prior to problem-solving, we provide a simple support task to help participants as they approach the problem. The more theoretical issue is our unique claim about the kind of representational content that is critically missing from default problem encodings. A rising viewpoint in cognitive science highlights the central role of relational cognition in human thought (e.g., Doumas et al., 2008; Gentner, 1983, 2003; Gentner et al., 2001; Gentner & Kurtz, 2005; Halford et al., 2010; Hofstadter & Sander, 2013; A. B. Markman & Stillwell, 2001; Penn et al., 2008). Any time we encounter a stimulus or situation that possesses some degree of complexity, there is the potential to encode what we experience not just as parts and properties, but also in terms of how these elements relate to each other: spatially, temporally, causally, or in terms of any relational predicate (such as actions like *chases* or relational categories like *obstacle* that we employ to assign relational meaning to the world).

A clear issue is that there would tend to be a very large (or infinite) number of possible relations that could hold within a stimulus or between a stimulus and other concepts. Further, it is presumably costly in terms of cognitive resources to invoke and evaluate this relational content (see Forbus et al., 1995). The relational reinterpretation hypothesis states that perceptual information can be reinterpreted in relational terms by humans, but not other species (Penn et al., 2008). Such reinterpretation is a capability of humans, but when not realized, it renders our cognition closer to that of other animals. Gentner (1988) has proposed a relational shift hypothesis which says that encoding stimuli in terms of structured representations is a matter of cognitive development (see also the representational redescription account; Karmiloff-Smith, 1990). These viewpoints all suggest a baseline in children and animals that people can transcend, but the notion that we are cognitively miserly (e.g., Stanovich, 2009) suggests that we might only do this work when required.

A good case in point comes from Markman and Gentner’s (1993) findings that participants tend to align the elements of two visual scenes by superficial similarity unless they are asked to engage comparison processes by evaluating the similarity of the scenes—in which case they become more likely to align the elements by their structural roles. In further support of the idea that default representations may be relationally impoverished and that benefits accrue from enhanced relational representation, the analogical processing literature shows that comparing analogs serves to highlight relations and facilitate the performance of cognitive tasks, such as understanding an unfamiliar domain (Kurtz et al., 2001), generating explanations (Hoyos & Gentner, 2017), and detecting commonalities and differences (Gentner & Gunn, 2001; Kurtz & Gentner, 2013; Markman & Gentner, 1993). Clement et al. (1994) provides a compelling example of the positive effect of relational content being manifest as opposed to latent in a study of analogical retrieval.

Our claim is that people should be capable of more effective and sophisticated cognition by applying standard information processing on more relationally rich representations. This is quite different from prior work linking creativity and analogical thinking (e.g., Holyoak & Thagard, 1995; Jones & Estes, 2015; Solomon, 1994), where much of the connection flows from the idea that bringing the right knowledge to bear can help one see something in a new way or solve a problem (see a review in Loewenstein, 2010). There is a well-known literature on analogical problem-solving and transfer that addresses the difficulty of spontaneous retrieval of source analogs (see Barnett & Ceci, 2002; Gick & Holyoak, 1980; Reeves & Weisberg, 1994; Ross, 1987) and the positive impact of comparing analogs during the study phase (Gick & Holyoak, 1983; see review by Alfieri et al., 2013) or at the time of test (Gentner et al., 2009; Kurtz & Loewenstein, 2007). To be clear, our current line of investigation links problem-solving and relational cognition in a different manner.

In the present work we are interested in the role of relational encoding for problem-solving independent of retrieval of analogous cases from memory. Our experiments involve the presentation of a novel insight problem without use of analogy-making to sources from a study phase or prior knowledge. Instead of relational encoding being a path to analogical retrieval, we address it as a path to escape functional fixedness. Accordingly, our research question is fairly straightforward: If we encourage participants to encode insight problems with a greater degree of relational encoding, will they be better at achieving the type of creative solutions that insight problems require? The claim is that people will be more successful in applying their reasoning capabilities toward creative problem-solving when their problem representation is more relationally rich.

Our technique for creating a more structured problem representation (i.e., more relational content) to promote insight problem-solving is novel, but the theoretical perspective has a precedent. Most broadly, Duncker (1945) recognized the important role of structural relations in the psychology of problem-solving (for a related discussion, see George & Wiley, 2019), and the notion of selective combination (Sternberg & Davidson, 1995) picks out the importance of connecting pieces of a problem representation toward insight. There is also a connection to Ohlsson and colleagues’ theoretical work (e.g., Knoblich et al., 1999) in terms of the roles of relaxing constraints and altering the organization of problem elements to overcome an impasse. Durso et al. (1994) report empirical evidence that problem-solvers become sensitive to the import of particular relations between problem elements as a precursor to restructuring and insight. Bieth et al. (2024) found that changes in semantic structure (such that concepts rated as semantically distant before problem solution were rated as more related after solution) are instrumental in analogical reasoning.

To draw out a comparison with McCaffrey’s (2012) focus on the functions of individual generic parts, the emphasis in relational encoding is on how the provided elements can interact. The possible convergence between these approaches is that the way problem elements interact may reflect a functional role; however, the core difference is also clear in terms of ascribed relations between given elements versus known functions of genericized elements. For example, in the famous two-string problem (see Table 1), the pliers are critical to solving the problem, as the tool can be tied to a string to create a pendulum effect (rather than using the tool for its usual purpose). On McCaffrey’s (2012) view, genericization of the pliers might reveal its potential role as a weighting element. Our view is that jointly evaluating pairs of problem elements (i.e., pliers and string) to consider their possible relationships is a particularly promising step. One can grab the string with the pliers (more conventional, less effective) or one can tie the string to the pliers (less conventional, more effective). Activating the latter relationship is likely to trigger further predication, or a mental simulation, of the weighted string swaying—in a manner that can be readily leveraged to solve the problem. Such relational encoding can lead to productive insights by inviting access to everyday knowledge that might otherwise be missed due to a bias to consider objects in isolation (or relative to goals). For example, thinking about a table and a book in relation to one another can activate content such as placing the book under a wobbly leg or reading a book for entertainment while eating alone. One specific way this can occur is by invoking the schema knowledge around the concept of one problem element as the basis to suggest novel analogical extensions for the other.

**Table 1.**
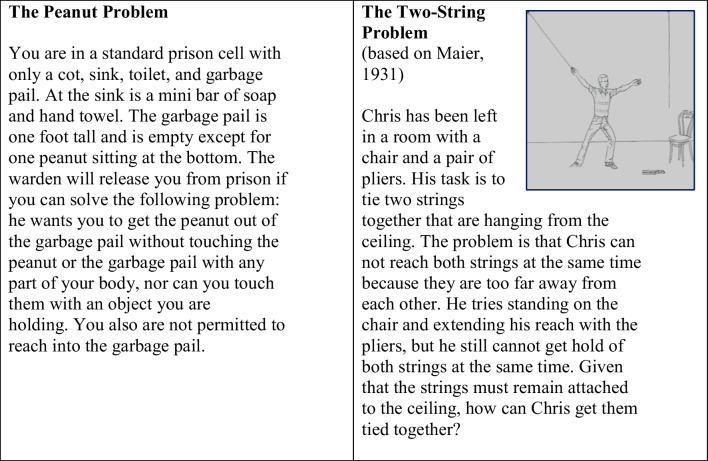
Materials used in Experiment 1

As noted above, in the existing literature, relational encoding has typically been elicited by inviting the comparison of alignable cases. This approach is not particularly well-suited to the traditional task of solving a single insight problem, so we developed a new direct relational encoding technique that in essence asks: what are the relations? More specifically, participants select pairs of elements from the problem description and complete sentence frames by filling in how the two elements relate to one another. Our prediction is that participants who engage in this relatively minimal support task when approaching an insight problem will succeed at a higher rate than controls.

## Experiment 1

This study evaluates the impact of a brief support task provided during consideration of the problem to encourage relational encoding of the problem situation. We operationalize relational encoding in a novel manner through direct elicitation of relationships between problem elements by filling in sentence frames. The selection of materials was based on finding insight problems with a reasonable level of naturalism (see Cunningham et al., 2009) and a basis for problem difficulty arising out of functional fixedness (i.e., having to think about problem elements outside of their canonical roles). Toward these goals, we chose situational word problems (rather than visuospatial puzzles) and specifically sought problems that are not just a matter of avoiding a trick (i.e., information stated in a particular way to make likely a misleading interpretation of the facts at hand; see Cunningham et al., 2009).

A nuanced and critical design issue for this project is the nature of the control condition. On one hand, one would like to use a control condition that is as much like the experimental task as possible without the specific critical element of invoking relational connections between problem elements. This would help to address potential issues such as time on task and depth of processing. However, we opt against this approach for several reasons. First, we are firmly convinced that better outcomes on insight tasks are not driven by such factors: We have seen no evidence that simply spending more time or thinking more deeply (i.e., semantically) about the problem leads to greater success. Insight problem-solving comes from thinking differently, so we consider it a lower priority for the experimental design to address these factors. We have not seen it in the laboratory and know of no empirical support in the literature for time on task promoting success in insight-based problem-solving (see review, Chu & MacGregor, 2011); if anything, there is some evidence of incubation effects (i.e., that time *away* from the problem can be helpful; e.g., Sio & Ormerod, 2009).

We did conduct an experiment using a control condition based on encoding within-element attributes instead of between-element relations; however, it became clear that it was impossible to tell whether the attribute encoding task might actually be suppressing insight-based problem-solving rather than serving the intended function as a tight control. Therefore, we elected to employ a baseline-style control condition to address the question of whether a relational encoding support task was better than no support at all (i.e., simply being given the problem to solve). The advantage of a baseline control (no encoding task) is that it makes certain we are not comparing relational encoding to another task that produces unintended or even deleterious effects. The disadvantage of a cold baseline control is that the relational encoding condition participants generally have more to do and may spend more time on task. It is our belief that most candidate manipulations would fail to show an advantage over such a baseline—which is to say that “something” is typically no better than ‘nothing’ when it comes to creative problem-solving. In sum, we believe relational encoding could provide a viable, real-world basis for improving creative problem-solving, so a good starting place is to see how it compares to the standard situation of natural, unguided problem encoding. Accordingly, the predicted outcome of an advantage for relational encoding support task over a baseline with no encoding support task would be a notable finding.

### Method

#### Participants

Undergraduate students (*N* = 168) from Binghamton University participated in this study and received research participation credit toward a course requirement. Eleven of the subjects were dropped due to submitting joke responses or blank solutions. For all experiments, participants gave informed consent and the experiment was IRB approved. Sample size was set under the goal of testing each problem under each condition with a cell size (*n* =  ~ 40) typical for studies of this type.

#### Materials and design

Participants were randomly assigned to the relational or baseline encoding condition and randomly assigned one of the two possible insight problems to solve (see Table 1). All of the problems were presented fully in text form except for the *two-string* problem (pilot results indicated that participants often seemed to not fully comprehend the problem situation, so an illustration was added to supplement the verbal description).

#### Procedure

The experiment was completed as one of the studies included in either a 30- or 60-min experimental session. In each condition, participants received (all on paper) experiment instructions, the insight problem, the encoding support task (if given), and an area in which to write their solution. The entire task was self-paced. At the top of the page was the problem, followed immediately below by the encoding task (if given), and then a solution space to describe their solution to the problem. Participants in the relational condition were asked to “come up with different ways in which the above objects could relate to one another (i.e., how they could interact, be used together, or be applied to one another).” The 10 relational sentence frames were as follows: “________ can relate to ________ in the following way: ________.” In the instructions, participants were asked to include every object from the problem, and it was noted that some objects may relate to one another in more than one way (allowing more than one answer using the same pair of objects). Participants were left with considerable flexibility in terms of how much to think about the problem during the support task, what the problem elements were, how many relations to provide for a given pair, whether to include all possible pairs, and whether or not to use all 10 response frames. We considered the possibility of bias (even with random assignment) arising from participants having familiarity with the two-string problem due to its presence in textbooks. In order to address this, we included a posttask familiarity questionnaire.

### Results and discussion

According to our exclusion criteria, four participants were dropped due to experimenter error, and 17 participants were removed for self-reporting familiarity with the two-string problem when prompted. Correct solutions conformed to the following rubric (note that no satisfactory solutions were found other than the expected ones). A response to the *two-string* problem was correct if the solution indicated attaching pliers to the string and swinging the string so that it gained momentum and became close enough to the other string to tie the two together. A response to the *peanut* problem was correct if the solution indicated transferring water with cupped hands or by saturating the hand towel and releasing water into the bucket to make the peanut float to the top. Two independent expert evaluators scored each response as correct or incorrect. There was one scoring discrepancy that was resolved through discussion. Hit rates by condition and problem can be seen in Table 2. Participants who completed the relational encoding task showed a 43% success rate which was reliably better than the 22% success rate of the baseline group with no encoding task, χ^2^(1, 136) = 6.585, *p* = 0.010, φ = 0.22.

**Table 2:** Experiment 1 hit rates (hits/total) by condition and problem

	Baseline	Relational encoding
Peanut	9/35 = 26%	15/34 = 44%
Two-string	6/33 = 18%	14/34 = 41%
Total	15/68 = 22%	29/68 = 43%

With regard to possible item differences, the differences in proportion correct between the control and the relational conditions were consistent (0.18 and 0.23). On the two-string problem, the difference was reliable—even with the substantial reduction in power that comes with subsetting the data by problem, χ^2^(1, 67) = 4.23, *p* = 0.039, φ = 0.25. For the peanut problem, the difference between relational and baseline groups approached significance but was not reliable, χ^2^(1, 69) = 2.57, *p* = 0.109, φ = 0.19. A secondary analysis was conducted to determine whether identifying a critical relation enhanced the likelihood of successfully solving the problem. For the *two-string* problem, the critical relation was that the pliers can be tied to the string. For the *peanut* problem, the critical relation was that the toilet or sink can be used to saturate the towel with water (note that the prisoner’s hands may not have been perceived as a problem element). It was found that 77% of participants in the relational group who successfully solved the test problem generated the critical relation during the encoding task, while only 22% of participants who got the problem incorrect generated the critical relation.

Subsequent to primary data collection for this project it was suggested to us that, despite the common intuition that peanuts float in water, it is not true under all circumstances. To be cautious, we re-ran the relational encoding condition with no changes, except we replaced the peanut with an object that consistently floats (a wooden coin). We were fortunate to have access to a larger sample (*n* = 77) from the same population, and the results were highly consistent as we once again observed a 44% solution rate.

## Experiment 2

This experiment was designed to further address the relational encoding advantage found in Experiment 1 (E1) in several ways. First, we sought to assess whether the relational encoding advantage would be conceptually replicable. Toward this goal, we chose to test relational encoding again but using a new problem to show that our findings are not limited to the particular materials employed. We selected an insight problem (Hot Coals; adapted from McCaffrey, 2012), which accords with the desired problem type in that it does not involve something designed to trick the solver but does require figuring out how problem elements can be brought together in nonobvious ways to generate a solution.

Secondly, we sought to address the limitation of only having evidence relative to a cold baseline. While it is sensible and translationally relevant to see whether the support task is better than nothing, it would be better to have stronger evidence that it is specifically the relational encoding that is driving the results. Lacking a definitively satisfying choice for a nonbaseline control condition, we adopted a two-pronged approach. First, we developed a simple control condition that involves studying the content of the problem toward being able to complete fill-in-the-blank memory probes. This helps to address the limitations inherent in the cold baseline of E1, but it is still not ideal in that we would not argue that it makes equivalent information processing to relational encoding, only that it is somewhat more comparable. In addition, we had in mind an idea for another type of support task for insight problem-solving that involved encoding context-dependent properties (Barsalou, 1982) of problem elements as opposed to encoding relations between them. This support task was deemed worthy of investigation in its own right but also offered potential utility for our follow-up experiment because it makes comparable information-processing demands as the relational encoding support task from E1 but without the key focus on relational content. Therefore, we included a third condition in the experimental design with the potential to serve one of two possible functions: 1) If the new support task was effective, we would have evidence of another promising path to improve insight problem-solving, and 2) if the new task did not succeed, it could serve as a de facto control by showing that a support task comparable in information-processing demands to relational encoding did not have the same facilitative effect. As a potential control, the context-dependent (CD) feature encoding task is as good as any alternative we could imagine—and perhaps better in the sense that it is grounded in a theoretically compelling basis for potential success (as described below). To summarize, our core prediction is an advantage of relational encoding over the memory control, and we include an exploratory CD support task that could also be effective—or could provide useful supportive evidence for the efficacy of relational encoding if it turns out that we replicate the relational encoding advantage over the memory control while the CD task with comparable information-processing demands provides no such advantage.

The new support task was inspired by Barsalou (1982), who proposed and offered empirical support for a psychological distinction in concept representation between context-dependent and context-independent properties; this was a precursor to his well-known work on ad-hoc or goal-derived categories such as *things to take out of the house in case of fire*. The basic claim is that knowledge of ordinary entity concepts is grounded in sets of context-independent (CI) properties that are things we expect and that come to mind when invoking a concept; in addition, there are context-dependent (CD) properties which are things we know about concepts that are only expected or activated under particular contexts. A canonical example is knowing that basketballs are round and bouncy (CI properties), while the knowledge that basketballs float is a CD property because it only comes into play under special circumstances (i.e., the ball went over the park fence and is rolling down toward the pool). Similarly, *fits in a suitcase* may not readily come to mind for a flashlight until packing (Barsalou, 1982). There has been no effort, to our knowledge, to relate this distinction to creativity, but we propose that encoding problem elements by thinking about their CD properties could be a path to better insight-based problem-solving. A problem may be difficult and require insight because the CI properties of the problem elements are not enough to find a solution. Activation of context-dependent properties can be impeded by the activation of context-independent properties which tend to be accessed regardless of setting (e.g., fixating on the edibility of an apple may prevent its use as a way to keep a book open). A focus on context-independent properties that interferes with the activation of context-dependent properties needed to solve a problem is therefore closely akin to functional fixedness that prevents problem solution (Duncker, 1945), except it is broader than just function since it potentially includes all the ways that one does not typically think about something but that one could with the right contextual cue. Recalling the example introduced above, of a book and a table as elements in a problem, we noted that it would be typical to consider standard properties of books and of tables as well as nonstandard properties of books and of tables that are activated by the specific goal context of the problem. The present idea goes another step by promoting the activation of nonstandard properties of books and of tables relative to arbitrary, self-generated contexts. For example, thinking of a table in the context of an airplane might activate the idea that a table can swing into place from a tucked-away position in a chair; thinking of a book in the context of house-cleaning might activate the tendency of books to collect dust.

It is helpful to consider how a CD/CI support task that involves generating context-dependent properties for problem elements compares in principle with relational encoding or the generic parts technique (GPT; McCaffrey, 2012). The main difference from relational encoding as implemented in this investigation is whether one is asked to think about relationships between pairs of problem elements or to think about properties of each problem element relative to self-generated contextual cues. Both can seemingly serve to activate ways of thinking about the problem elements that are nonstandard and that could lead to an insight-based solution following from the activation of atypical semantic content. Context-dependent properties will not necessarily access the relational structure, although some context-dependent properties activate relational properties. To use a chair as a doorstop (Barsalou, 1982), one must activate or otherwise infer the context-dependent property of a chair “being able to prop open a door” and activate the relational category of “items able to prop open a door” (see Gentner & Kurtz, 2005; A. B. Markman & Stilwell, 2001, for discussion of relational categories). McCaffrey (2012) also invokes the idea of activating nonstandard properties of problem elements, but instead of searching for CD properties (i.e., what is true of the concept but only comes up in a specialized context), the approach is to conduct a systematic but not context-driven process of identifying semantic elements that may not come immediately to mind. While previous work has found that directing attention toward context-independent properties hinders problem solution (Duncker, 1945; Schooler et al., 1993), and directing attention away from context-independent properties (Duncker, 1945; Glucksberg & Danks, 1968) or toward an obscure property via GPT (McCaffrey, 2012) promotes problem solution, we do not know of any work examining the effectiveness of directing attention specifically toward context-dependent properties.

### Method

#### Participants

A total of 185 undergraduate students from Binghamton University participated in the experiment for partial fulfillment of a psychology course requirement. A larger sample (~ 60 per cell) was targeted in this experiment in anticipation of applying subject exclusions based on failure to satisfactorily perform in the support tasks (and to thereby get a cleaner result based on assessing the problem-solvers in each condition who carried out the support task as intended).

#### Design

Participants were randomly assigned to either the Control, Relational Encoding (RelEnc), or Context-Dependent/Context-Independent (CD/CI) conditions of a between-subjects design.

#### Procedure

We opted to employ a training phase to help ensure that participants accurately comprehended and could carry out the tasks critical to the experimental manipulation. This involved practicing the task (identifying relations or generating context-dependent properties) with content unrelated to the actual test problem. After the training (or right from the start in the case of the Control condition, which required no training), participants completed a condition-specific support task and then the actual problem-solving activity (the latter was the same across all conditions). Details of the phases specific to each condition are provided below.

#### Training phase

##### Relational Encoding (RelEnc) 

Participants received instruction via Qualtrics on how to properly generate relationships between object pairs (e.g., how the objects may interact, be used together, or apply to each other). They were shown examples of well-formed relational responses between the pair *sun–sunflower* (e.g., the sun shines on the sunflower or provides it energy, and the sunflower grows toward the sun) and incorrect responses (e.g., both are yellow or contain the word *sun*). The training required participants to identify the relationship between four object pairs: *engagement ring–popcorn, book–spoon, mask–bubble gum, umbrella–vase*. These items were intended to be irrelevant to the insight problem in the actual experimental test. For each pair, participants were shown three options and asked to select the best description of a relationship between the objects. For example, when shown the pair *engagement ring–popcorn*, participants could select either 1) The items are the same size, 2) The ring could be buried beneath the popcorn, or 3) Someone eating popcorn could be wearing an engagement ring. The correct answer for each training item was provided regardless of accuracy. Participants were again informed that correct responses described how the objects could be used together, interact, or apply to each other; incorrect selections merely described a sequence of events that included the objects or features shared between objects, but did not describe a direct action or impact that could occur between the objects.

##### Context-Dependent/Context-Independent (CD/CI)

Participants were shown, via Qualtrics, a description of context-dependent and context-independent properties (adapted from Barsalou, 1982). Context-independent properties were described as those that are typically associated with the object, regardless of context, and come to mind easily (e.g., an object’s color or typical use). Context-dependent properties were described as those that become apparent only in a specific context (e.g., one may not consider whether an object can be used as a lever unless there is a situation that requires additional physical force). Participants were asked to generate (via textbox input) one to five context-independent and one to five context-dependent properties of a pencil (an object irrelevant to the test problem). After completion, participants were given feedback in the form of a list of good responses for the context-independent (e.g., has a red eraser, made of wood) and context-dependent (e.g., floats in water, can fasten a hair bun) properties of a pencil.

#### Support task phase

##### Control 

The control group was given (via Qualtrics) a memory task based on the Hot Coals insight problem. The goal was to create a broad degree of equivalence in task experience and content exposure relative to the experimental conditions. The memory task was chosen as a means of achieving this type of control while being unlikely to create a positive or negative effect on subsequent problem-solving performance. One imbalance that arose was that the control group had the advantage of repeatedly seeing the test problem during the support task phase prior to the actual problem-solving phase, while the experimental groups only saw keywords from the test problem (this advantage works in the favor of the Control condition so does not undermine any effects observed relative to the Control). As for the specific procedure followed, participants were initially shown the insight problem to read before a series of fill-in-the-blank-style questions. For each of four trials, the entire insight problem was shown with one problem-relevant word (*volleyball, rocks, sand, burn*) missing. Participants were asked to recall the missing word and type it into the textbox provided. Feedback was not explicitly provided, but the subsequent fill-in-the-blank question would display the insight problem with the previously missing word in place.

##### Relational Encoding (RelEnc) 

**Fig. 1:**
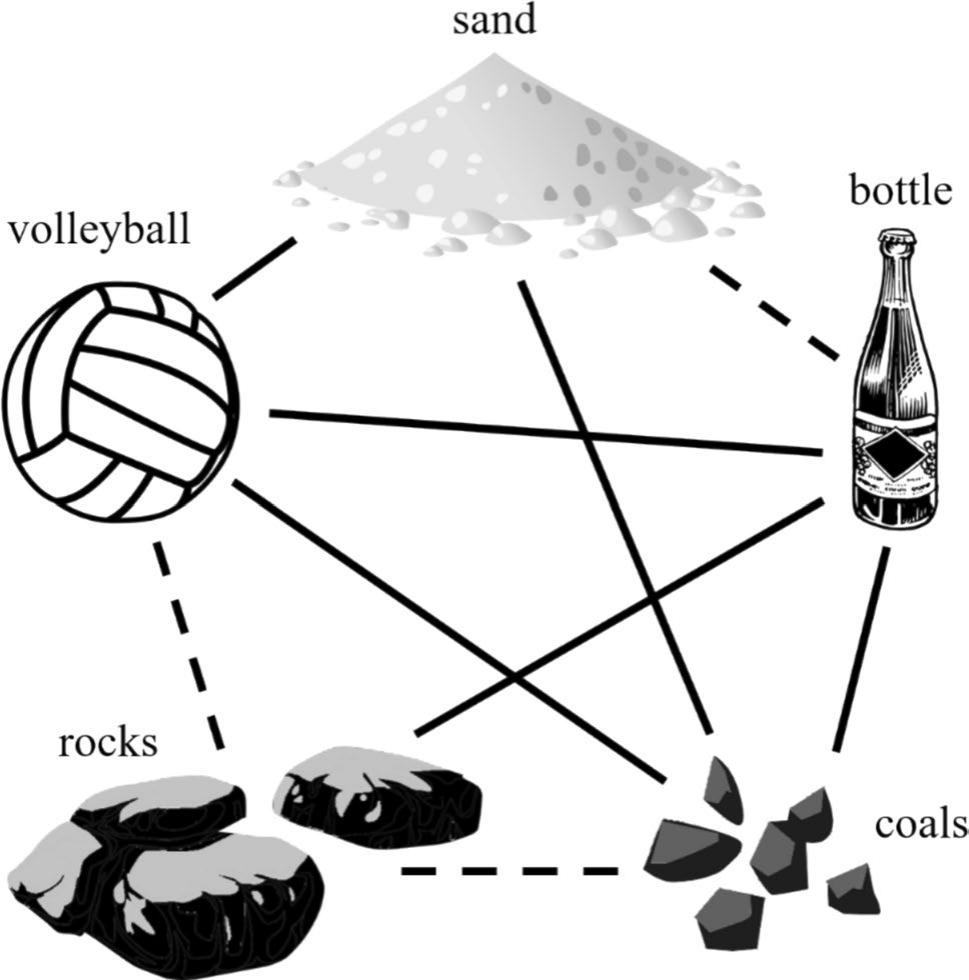
Relational diagram of problem-relevant objects in Experiment 2. The relational encoding condition asks for possible relations between each of the five problem-relevant objects connected by lines. Solid lines show relations that contribute to an Ideal solution

After completing training, participants were shown the insight problem but not yet prompted to provide solutions. Using pen and paper, participants were asked to apply the technique on which they had just been trained—they were asked to generate relations (at least one for each) between the following pairs of problem-relevant objects (*volleyball–coals; volleyball–bottle; volleyball–rocks; volleyball–sand; coals–bottle; coals–rocks; coals–sand; bottle–rocks; bottle–sand*; a diagram of relations between the problem-relevant objects is shown in Fig. 1). Participants were given three blank lines per object pair to write a relation. No feedback was provided.

##### Context-dependent/context-independent (CD/CI)

Using pen and paper, participants were instructed to list one to five context-dependent properties for each of the problem-relevant objects (*rocks, bottle of beer, volleyball, hot coals, sand*) before having seen the actual insight problem. Participants were not shown the problem at this point because it was found in pilot testing that participants would often restrict their responding to the particular problem context. No feedback was provided.

#### Test phase: Insight problem-solving

After completion of the training and support task phases, participants were given a new worksheet with instructions at the top of the page stating: “Now you’re ready to move on to the next part of the experiment and answer The Hot Coals Problem.” The problem was adapted from McCaffrey (2012) and presented as follows:

You have been enjoying a campfire on the beach when you suddenly remember that campfires are only allowed in a designated area. You have no additional coal or matches, so you decide to try to quickly transport the hot coals to the legal area about 100 yards away. The coals are much too hot to touch, and they would quickly burn through any cloth/clothing they come in contact with. You are barefoot, wearing only a swimsuit, and all you have with you is a beach volleyball and a bottle of beer. The area is barren except for some small rocks around the campfire and the surrounding sand. How can you move the hot coals all at once before they cool?

Adaptations were made from the original version toward the goals of making the problem statement and solution more natural and to help make tempting but unsatisfactory responses less likely. Specifically, we provide a rationale for moving the coals, make clear that there are no matches or additional coal available, specify being clothed in a swimsuit (which is appropriate to the beach and discourages solutions involving made-up clothing items), and switch from the original basketball to a volleyball (more appropriate to the beach and more clearly amenable to being cut open). The problem was presented on paper with empty space on the page where participants were instructed to provide their handwritten solution as follows: “In the space provided below, please describe your solution ideas to The Hot Coals Problem. Don’t be vague, be sure to describe your solution to The Hot Coals Problem thoroughly!”.

### Results

#### Support task scoring

For all of the conditions, the support task (fill-in-the-blank for Control, generating context-dependent and context-independent properties for CD/CI, and generating relational categories for RelEnc) was scored for accuracy and evaluated relative to exclusion criteria. Control participants were required to produce error-free support task responses, and, as a result, six participants with errors were excluded from further analyses. One of the four fill-in-the-blank questions had the correct answer [surrounding _sand_], however [surrounding _area_] was also accepted as a correct answer given that it is a natural expression in English that effectively captures the intended meaning.

For the remaining conditions (RelEnc, CD/CI), expert scorers judged the pen-and-paper responses for being task compliant (each was evaluated by one scorer, but discussion was used as needed). Participants had been instructed to provide at least one context-dependent property (CD/CI) or object-pair relation (RelEnc) for each prompt shown during the second phase of training; however, there was no set limit on the amount of answers participants could provide per prompt. As such, percentage of accuracy for each participant was calculated out of the total answers given. Skipped prompts, nonrelational responses for object pairs (RelEnc), or context-independent properties listed rather than context-dependent properties (CD/CI) were considered incorrect. In most cases, the accuracy was either excellent or quite poor, but a 50% accuracy cutoff was used to provide a definitive basis for exclusion. Based on these criteria, 13 participants were removed from the CD/CI condition and eight from the RelEnc condition. An additional 13 participants were removed due to software or logistical difficulties that prevented completion; this resulted in a final tally of Control: *n* = 54; RelEnc:* n* = 49; and CD/CI: *n* = 42.

##### Insight problem scoring

 Each response was scored by an expert rater blind to condition. Scoring was based on a rubric that detailed scoring criteria and defined scores for specific common solutions. Ambiguous items were resolved by discussion between scorers. Responses were scored in two ways. The first assessed the solution quality (“‘Ideal” solution, “Complete” solution, “Partial” solution, “No” solution) and represented a departure from a basic success/failure dichotomy under the rationale that there are potentially meaningful nuances in solution quality that are lost under binary outcome scoring. This scoring method differs from E1 in part because the previous scoring experience left the research team with a concern that potential benefits of relational encoding may not register in the results when an entirely correct response was not achieved. Additionally, the Hot Coals Problem requires a coordinated set of multiple insights, so it is particularly important to credit cases that fell short of a completely successful solution.

An “Ideal” solution mentioned the use of all problem-relevant objects: breaking the bottle on the rocks, using the sharp ends to cut open the volleyball, filling the ball with sand (to insulate the coals), and using part of the bottle to scoop all the hot coals into the ball. A “Complete” solution did not mention all ideal elements but remained recognizable as the same solution (e.g., a response that mentions breaking the bottle but does not mention that the bottle was broken on rocks). A “Partial” solution involved transporting only some of the coals successfully, not moving all coals simultaneously, or manipulating the coals invalidly (e.g., cooling the coals before moving, or moving the coals in a way that would burn the participant). A response considered a “No” solution was any that would not work at all (e.g., pushing coals across the sand with your hands). The second scoring approach assessed the creativity level of the response. Two creativity points were awarded for the first unconventional use of a problem-relevant object; one point was awarded for each subsequent use of that object. For example, drinking out of the bottle is conventional, so no points would be awarded. However, using the bottle shards to cut the volleyball would be considered unconventional and awarded two points; the subsequent use of the broken bottle to scoop coals would be awarded one point. No points were considered for nonproblem relevant objects (e.g., ocean water, beer in bottle, swimsuit).

##### Insight problem-solving outcomes

 The frequency of each type of insight problem solution (Ideal, Complete, Partial, No) was evaluated between the Control, Relational Encoding (RelEnc), and Context-Dependent/Context-Independent (CD/CI) conditions to determine whether exposure to the different training tasks lead to a different rate of solution types. A chi-squared test for goodness of fit (using control proportions as expected values) revealed a significant advantage for RelEnc over the Control group, χ^2^(3, *n* = 103) = 9.18, *p* = 0.03, Cramer’s V = 0.30, but no difference between the CD/CI and Control groups, χ^2^(3, *n* = 96) = 0.32, *p* = 0.96, V = 0.06. As can be seen in Table 3, the RelEnc condition had a higher rate of ‘Ideal’ and ‘Complete’ solutions than the CD/CI or Control groups; and likewise RelEnc had lower rates of ‘Partial’ and ‘No’ solutions than the CD/CI or Control groups. The outcome of a relational encoding advantage without a CD/CI advantage relative to the Control group provides a valuable conceptual replication of the relational encoding benefit with a stronger (memory-based) control in place than the cold baseline of E1. The failure to observe a benefit of CD/CI support task suggests that this is not a promising approach, at least as implemented, to improve insight problem-solving. Additionally, the failure of the CD/CI support task serves to reinforce the case for relational encoding—given that another comparably sophisticated and demanding support task did nothing to improve problem-solving outcomes. In combination with the use of the memory control, this counts as considerable evidence that the efficacy of the relational encoding support task can be attributed to its specific quality of activating relationships between problem elements. A secondary statistical analysis directly comparing the RelEnc and CD/CI conditions (using the CD/CI proportions as expected values) gives a degree of additional support for this conclusion in the form of a marginal trend favoring relational encoding, χ^2^(3, *n* = 91) = 7.44, *p* = 0.06, V = 0.29.

**Table 3:** Experiment 2 completion score percentages by condition

Conditions	Ideal	Complete	Partial	No
Control	9.26%	29.63%	42.59%	18.52%
CD/CI	9.52%	33.33%	40.48%	16.67%
RelEnc	20.41%	34.69%	32.65%	12.24%
Total	13.10%	32.41%	38.62%	15.86%

The relational encoding (RelEnc) condition showed more Ideal and Complete scores, and fewer Partial and No scores, than the other two conditions. A chi-squared test for goodness of fit found a significant difference between the RelEnc scores and Control scores, and not between CD/CI and Control scores

**Fig. 2:**
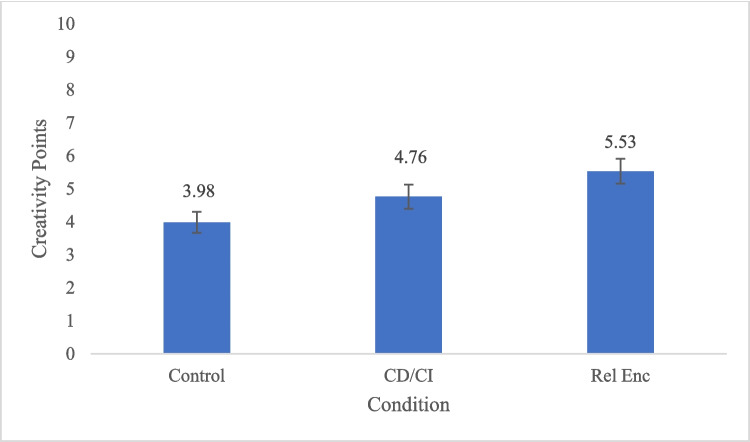
Mean creativity points per condition in Experiment 2. The Relational Encoding condition significantly outperformed the control in creativity points while the CD/CI condition did not. Error bars represent standard error of the mean

**Fig. 3:**
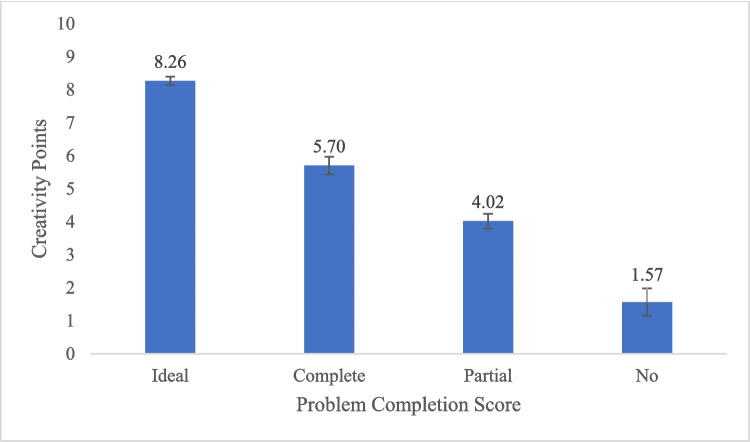
Mean creativity points per problem completion in Experiment 2. Each better completion score received more creativity points on average than worse completion scores. Error bars represent standard error of the mean

A one-way ANOVA revealed a significant difference in creativity points between conditions, *F*(2,142) = 5.00, *p* = 0.008, η^2^ = 0.066, with a small effect size. Overall, mean creativity points per condition showed an increase in problem-solving creativity from Control (*M* = 3.98, *SD* = 2.38) to CD/CI (*M* = 4.76, *SD* = 2.41) to RelEnc (*M* = 5.53, *SD* = 2.66) conditions (see Fig. 2). While these were planned comparisons, we conservatively applied Tukey–Kramer post hoc tests, which revealed a significant difference between the RelEnc and Control conditions, *q* = 4.47, *p* = 0.005, but not between CD/CI and Control, *q* = 2.16, *p* = 0.28, or between the CD/CI and RelEnc and conditions, *q* = 2.08, *p* = 0.31. Both primary analyses support the conclusion that relational encoding, uniquely among the tested conditions, facilitated the emergence of creative insights during problem-solving. We also expected a relationship to hold between the two scored measures; Fig. 3 illustrates how Ideal solutions had the highest average of creativity points (*M* = 8.26, *SD* = 0.56, *n* = 19), followed by Complete (*M* = 5.70, *SD* = 1.82, *n* = 47), then Partial (*M* = 4.02, *SD* 1.67, *n* = 56), and lastly responses with No solution had the lowest average number of creativity points (*M* = 1.57, *SD* = 2.00, *n* = 23).

In addition to performance, it is worth considering aspects of response latency. In a cognitive task such as creative problem-solving, there is little reason to believe that just spending more time will have a substantive impact on performance. We intentionally did not design the procedure to equate time spent on the support tasks or the core problem-solving task across conditions. It is worth noting that the RelEnc support task took longer to complete than CD/CI and both took longer than the memory task of the Control. Our research question addressed the impact of properly carrying out the support tasks rather than the time it took, so we cannot rule out the possibility that the time required by the support task contributed to some degree to the outcome. For a subset^1^ of participants (*n* = 78) we recorded response times for the problem-solving task itself (noting that the RelEnc and Control groups had access to the full problem before actually being asked to solve it and the CD/CI group had access to semantic elements of the problem removed from the problem context). In addition to the aforementioned data exclusions, data from six participants were removed specifically from this analysis due to having an outlier RT (durations greater than 11 min, as determined by interquartile range from the third quartile; no lower outliers were present), resulting in *n* = 50. Average problem-solving duration expressed in mm:ss ranged from RelEnc (*M* = 06:37, *SD* = 02:02, *n* = 18) to CD/CI (*M* = 06:12, *SD* = 02:27, *n* = 15) to Control (*M* = 04:56, *SD* = 01:47, *n* = 17) conditions. A one-way ANOVA revealed no significant difference for problem-solving duration between conditions, *F*(2,47) = 2.98, *p* = 0.06; however, this does suggest a marginal trend for participants completing the more sophisticated support tasks to spend more time subsequently working on the problem. This could reflect greater engagement in the problem-solving task for these conditions, but notably, although the CD/CI and RelEnc groups spent similar amounts of time, they had divergent problem-solving outcomes. On the question of how individual response times relate to performance outcomes, we found that those with higher creativity point scores spent longer solving the problem, *r*(48) = 0.40, *p* = 0.004. An analysis across the levels of solution quality (“Ideal” being the best and “No” being the worst) did not show a significant difference in time spent on the problem, *F*(3,46) = 1.64, *p* = 0.19.

In sum, this experiment provides considerable additional support using a new test problem for the facilitative effect of relational encoding found in E1. Significant advantages are observed in the dependent measures of problem-solving outcome and evidence of creativity relative to a memory task control (as opposed to the cold baseline of E1). In addition, these positive results occur in conjunction with the failure of an ostensibly promising manipulation (CD/CI) that makes similar information-processing demands to show an improvement relative to the memory control. Relational encoding shows a mix of reliable advantages or marginal trends when analyzed directly against CD/CI as a de facto control. This pattern of findings constitutes a compelling set of evidence supporting the hypothesized relational encoding advantage. We take up further theoretical and applied implications of finding a relational encoding advantage (and no CD/CI advantage) in the general discussion.

## General discussion

Unlike existing research, in which relational structure plays a critical role as the basis for problem-solving via analogical retrieval, we investigate the importance of relational encoding in setting up a problem representation that invites overcoming functional fixedness. Further, we introduce a task to promote relational encoding without requiring comparison to another analogous case based on straightforward prompting to identify relations that hold for pairs of problem elements. The finding of note is that successful insight problem-solving rates with relational encoding support are significantly higher than a cold baseline (E1) and a memory task control (E2); further, the relational encoding condition showed a reliable advantage over the memory control while a comparably demanding and theoretically viable alternative support task (CD/CI) had no facilitative impact. The latter finding is also of interest given that previous work has found that pulling participants’ focus away from typical item properties promotes successful problem-solving (Duncker, 1945; McCaffrey, 2012), while we found that highlighting nontypical, context-dependent information did not promote successful problem-solving.

The core finding of a relational encoding advantage is demonstrated across two experiments, providing a conceptual replication with a promising degree of robustness over materials, methodological details, and control conditions. While the results as they stand are notable, we consider it possible that a stronger version of the manipulation could produce further boosts to performance by 1) more rigorously guiding participants to posit and evaluate relationships among problem elements; 2) more overtly linking the support task to the actual problem-solving task (nothing was done to specifically encourage participants to leverage the results of the encoding task in their actual problem-solving effort); 3) contextualizing and motivating the relational encoding support with an explanation of the issue of functional fixedness; or 4) turning the relational encoding support into a more comprehensive training. Such proposed modifications would compromise the minimalism of the intervention to some degree, but not overly so.

A more skeptical view of the results can also be taken. Many participants still failed to solve the problem, so this is not a guaranteed recipe for problem-solving success. Also, we do not know how important the training phase was to performance in E2; it does seem that people do not always make the right interpretation of what we mean by relationships between problem elements. There are many other examples and varieties of insight-based problems that are not addressed here, so we do not yet know the breadth of impact of relational encoding. It does seem likely in our view that many insight problems are amenable to relational encoding insofar as 1) having a set of identifiable problem elements to relate and 2) having a solution that becomes more visible as a result of activating relations between problem elements. There may be problems that require overcoming fixedness about a single object such that relations between problem elements do not really factor in. As a thought exercise we consider an example of experiencing a severe leg injury and needing to find a way to cover ground; a solution may be figuring out a makeshift crutch (perhaps a kitchen broom?). This seems driven by a recognized need for a crutch substitute and identifying a latent similarity between a crutch and a broom—however, a possible solution path could arise from invoking relationships between one’s body and the broom that activates a notion of leaning on or using the broom for support. The conventionality of the crutch solution is perhaps the reason that a single-object approach seems more likely in this case.

The observed relational encoding advantage suggests that directing focus toward a more relationally rich construal helps to overcome functional fixedness and promote insight-based problem-solving. A relevant distinction lies between preventing fixedness from setting in initially versus finding a way to escape from it. We do not take a strong stand on this issue in part because we see problem encoding as flexible and evolving. That said, our expectation is that relational encoding fits better with a framework of restructuring the problem representation than preventing a conventional view of the problem elements from arising at all. The present findings expand on McCaffrey (2012) by demonstrating that a novel problem-solving method focused on relational content is successful in solving an insight problem. This finding also suggests that directly guiding attention away from context-independent features of individual problem elements need not be the predominant route to overcoming functional fixedness (Duncker, 1945). The effect of invoking between-element relations may arise in part due to what the thinker moves away *from* as well as what they move *toward*.

The primary theoretical advance in the present work is that insight problem-solving can be improved by a route that does not focus on changing how people think about individual objects in the problem independently, but instead on meaningful ways for the objects to interrelate. The notion of a swinging string is not arrived at in these experiments by avoiding standard features and landing upon an obscure feature of the string, but by activating a relatively evident consequence that arises from interrogating two objects as a relatable pair: the conjunction of a hanging string and a small, weighted object reveals the relation of attaching them along with contingent impacts of a hanging relationship between the objects. The critical cognitive piece is what one is able to think of when thinking about two objects together. The pliers are not a cue to activating the swinging property of the string without invoking the relational content. As a thought exercise, imagine a question like this: what properties come to mind about a string given the cue of pliers or about pliers with the cue of string? We argue that it is not the mere juxtaposition of the internal semantics of the two objects, but the effort to relate them that drives toward a solution.

Our results support previous findings on the importance of relational structure in supporting advanced cognitive processes (e.g., Gentner & Asmuth, 2019) and problem-solving (see Goldwater & Schalk, 2016, for a review of relational cognition and education). The value of relational information is highlighted when examining problem-solving methods of experts and novices. Experts have more access to relational content than novices; for example, novices sort physics problems by types of items (superficial content) within the problems, while experts will sort by types of processes (relational content; Chi et al., 1981). Chi and VanLehn (2012) have investigated how experts access this relational content, and how novices can be taught to similarly access this content. They suggest that experts first access the relational information present between the specific items (superficial content) within a given problem. The authors recommend that students should be instructed to focus on finding the relationships between the items explicitly mentioned in the problem space.

Recent work demonstrating transfer of a relational mindset across different tasks (Chaxel, 2015; Vendetti et al., 2014; see also Kray et al., 2006; K. D. Markman et al., 2007) may also be consistent with this research direction. This in fact raises the question of whether the present findings can be interpreted in these terms: is the effect of relational encoding due to encouraging a relational mindset that promotes more relational thinking toward solving the problem (i.e., independent of the relational content explicitly activated in the encoding task)? We note that on this view one would seemingly have to predict that engaging in the relational encoding task for a totally unrelated problem would be equally effective—and this seems unlikely. Even so, promoting a relational mindset could be a contributing factor above and beyond the benefits of improving the encoding of the target problem.

While it was not our primary focus, the failure of the CD/CI condition merits further discussion. Previous work has shown that distracting away from context-independent features aids in the generation (and speed) of solutions to insight problems (Duncker, 1945). Failure to solve some insight problems has been said to lie in functional fixedness: a hyper-fixation on the most typical item uses or features which may then interfere with the activation of less typical, but more useful item features. The lack of success of the CD/CI condition contrasts findings from McCaffrey (2012). The generic parts technique has participants list the most basic features of items in the problem space, with no emphasis on context dependence or independence of the features—and participants with GPT training listed more uncommon item uses and were significantly more likely to solve insight problems. Our protocol differed from McCaffrey (2012) in a few important ways. The GPT repeatedly prompts participants to break objects into component parts until they cannot break apart the object any further or they arrive at a description that does not imply any specific use. CD/CI training used here taught participants about context-dependent properties and asked them to list a self-guided amount of properties before solving the problem. However, the GPT should spread focus to all (basic) object properties while CD/CI training should direct participants to only obscure or context-dependent features of items. This suggests that directing focus to only context-dependent properties, rather than away from context-independent properties (Duncker, 1945; Glucksberg & Danks, 1968) or toward basic properties (McCaffrey, 2012) is not sufficient to overcome functional fixedness and promote insight problem solution.

We conclude by considering broad theoretical and translational implications and directions for further research. The present work represents a new approach to fostering restructuring by prompting problem-solvers to engage in developing a more relationally rich problem representation. The theoretical implication is that shortcomings in creative cognition may be linked to a tendency toward overly sparse relational representations. In Guilford’s (1967) terms, one might say that encouraging connections between problem elements invites a divergent component that moves the thinker away from fixed and canonical consideration of each object and toward activation of novel relational content—as well as a convergent component that moves the thinker toward seeing a core relationship among problem elements that supports a singular solution. The takeaway from these results from a translational perspective is that simple supports to boost relational encoding during an insight problem-solving task lead to better solution rates—and it may be possible to encourage people via more robust training to develop habits of mind for applying this approach without requiring direct support.

An important theme of research in the psychology of problem-solving is the importance of the initial problem representation (i.e., how to break down the problem into its component parts or elements for consideration). This has been emphasized in simulation studies (e.g., Lovett & Forbus, 2017); behavioral experimentation (e.g., Duncan et al., 2017) and translational work (e.g., Cronin & Loewenstein, 2018). We elected to study problems that essentially “wear their elements on their sleeves” and, beyond that, we made the problem elements clearly identified to participants. Questions for future work include whether problem-solvers can apply relational encoding when they have to gather the elements themselves; or parse a problem description that does not lend itself to a clear breakdown into elements. In our view, there are many cases in which the elements are evident, and the present research suggests the value of relational encoding under such circumstances. We would consider the impact of relational encoding to be an elaboration of problem representation in the sense that the identified elements are made to participate in a richer relational milieu than problem-solvers would be likely to construct without the support task. As for the impact of relational encoding when the elements themselves are unclear, a few possibilities arise. In the case where an infelicitous parse is selected it seems unlikely that relational encoding would right the ship. On the other hand, it is possible to imagine that relational encoding could provide a pathway to a better parse to the extent that constructing relationships among a first draft of problem elements might reveal alternate breakdowns or combinations of information into problem elements.

In terms of real-world settings, people might have to operate in a more sensorimotor environment rather than text-based reading-and-writing to identify problem elements and relations—this could change how relational encoding works. Additionally, there may be emotional factors or time pressure that impact the problem-solving process. An important distinction is between mundane (yet potentially challenging) problems such as lost car keys or being stuck versus larger-scale, more global problems (e.g., how to save a failing business, clean up an oil spill, resolve urban blight). There seems to be a fairly good match between the type of problems in our experimental materials and mundane real-world problems, but it is less clear whether relational encoding could scale to more global problems. It is presumably more difficult to pinpoint the problem elements and to identify relations between them, but functional fixedness could still be in play. The search for solutions to global problems presents the greatest difficulty and reflects the highest stakes in human affairs, so any potentially promising avenue to progress is consequential. In such cases, relational encoding could work best as a tool for domain experts (rather than ordinary individuals) who might have the best chance at successfully articulating and relating complex problem elements in a fruitful manner.

## Supplementary Information

Below is the link to the electronic supplementary material.

Supplementary file1 (XLSX 127 KB)

## Data Availability

Data available as supplementary material and experimental materials included in manuscript.

## Footnotes

1 Data collection began without recording this information, but it was decided to begin doing so.

## Declarations

### Open practices statement

The stimuli for these experiments are provided in the manuscript and data are made available as supplementary materials. The experiments were not preregistered.
