# Correction: Relational encoding promotes creative insight for problem-solving

**DOI:** 10.3758/s13421-026-01913-2

**Published:** 2026-07-28

**Authors:** Kenneth J. Kurtz, Leif Haley, Alexus Longo, Shanti Astra, Hannah Meltzer, Gavin Suwara, John D. Patterson

**Affiliations:** 1 Department of Psychology, Binghamton University, PO Box 6000, Binghamton, NY 13902 USA; 2 Department of Psychology, Pennsylvania State University, State College, PA 16801 USA; 3 Department of Psychology, University of Wisconsin-Madison, Madison, WI 53706 USA

**Correction: Memory & Cognition**

10.3758/s13421-025-01685-1

The article “Relational encoding promotes creative insight for problem-solving”, written by Kenneth J. Kurtz et al. was originally published electronically on the publisher’s internet portal on 24 January 2025 without open access. With the author(s)’ decision to opt for open access, the copyright of the article changed on 13 June 2026 to © The Author(s) 2026 and the article is forthwith distributed under a Creative Commons Attribution 4.0 International License, which permits use, sharing, adaptation, distribution and reproduction in any medium or format, as long as you give appropriate credit to the original author(s) and the source, provide a link to the Creative Commons license, and indicate if changes were made. The images or other third party material in this article are included in the article's Creative Commons license, unless indicated otherwise in a credit line to the material. If material is not included in the article's Creative Commons license and your intended use is not permitted by statutory regulation or exceeds the permitted use, you will need to obtain permission directly from the copyright holder. To view a copy of this license, visit http://creativecommons.org/licenses/by/4.0/.

